# Myoferlin Regulates Wnt/β-Catenin Signaling-Mediated Skeletal Muscle Development by Stabilizing Dishevelled-2 Against Autophagy

**DOI:** 10.3390/ijms20205130

**Published:** 2019-10-16

**Authors:** Shunshun Han, Can Cui, Haorong He, Xiaoxu Shen, Yuqi Chen, Yan Wang, Diyan Li, Qing Zhu, Huadong Yin

**Affiliations:** Farm Animal Genetic Resources Exploration and Innovation Key Laboratory of Sichuan Province, Sichuan Agricultural University, Chengdu 611130, Sichuan, China; hanshunshun@stu.sicau.edu.cn (S.H.); cuican123@stu.sicau.edu.cn (C.C.); hehaorong@stu.sicau.edu.cn (H.H.); shenxiaoxu@stu.sicau.edu.cn (X.S.); chenyuqi@stu.sicau.edu.cn (Y.C.); as519723614@163.com (Y.W.); zhuqing@sicau.edu.cn (Q.Z.)

**Keywords:** myoferlin, myogenesis, autophagy, atrophy, Dishevelled-2

## Abstract

Myoferlin (MyoF), which is a calcium/phospholipid-binding protein expressed in cardiac and muscle tissues, belongs to the ferlin family. While MyoF promotes myoblast differentiation, the underlying mechanisms remain poorly understood. Here, we found that MyoF not only promotes C2C12 myoblast differentiation, but also inhibits muscle atrophy and autophagy. In the present study, we found that myoblasts fail to develop into mature myotubes due to defective differentiation in the absence of MyoF. Meanwhile, MyoF regulates the expression of atrophy-related genes (Atrogin-1 and MuRF1) to rescue muscle atrophy. Furthermore, MyoF interacts with Dishevelled-2 (Dvl-2) to activate canonical Wnt signaling. MyoF facilitates Dvl-2 ubiquitination resistance by reducing LC3-labeled Dvl-2 levels and antagonizing the autophagy system. In conclusion, we found that MyoF plays an important role in myoblast differentiation during skeletal muscle atrophy. At the molecular level, MyoF protects Dvl-2 against autophagy-mediated degradation, thus promoting activation of the Wnt/β-catenin signaling pathway. Together, our findings suggest that MyoF, through stabilizing Dvl-2 and preventing autophagy, regulates Wnt/β-catenin signaling-mediated skeletal muscle development.

## 1. Introduction

Autophagy, including macroautophagy, microautophagy, and chaperone-mediated autophagy, was first described in 1963 [1]. Autophagy refers to the encapsulation of cytoplasmic components, such as proteins and organelles, destined for transport to lysosomes for degradation [2]. Under stress conditions, such as starvation and hypoxia, autophagy is activated to promote cell survival by releasing energy substrates through degradation of cellular components and elimination of defective or damaged organelles [3]. It is now clear that the cell survival function of autophagy is an important evolutionary conservative mechanism by which eukaryotic cells maintain homeostasis and achieve renewal [4]. Skeletal muscle is composed of highly organized myofibers and its lean mass provides a tissue amino acid source that can be used under conditions of stress or starvation [5]. The ubiquitin-proteasomal pathway and the autophagic/lysosomal pathway are two highly conserved pathways mediating protein degradation in skeletal muscle [6]. In the ubiquitin-proteasomal pathway, ubiquitin-tagged proteins are degraded in the proteasome complex after conjugation with multiple ubiquitin moieties [7].

The turnover of most soluble and myofibrillar proteins in normal skeletal muscle is performed through the constitutively operative ubiquitin-proteasomal pathway [8]. Many studies have shown that autophagy is also constitutively active in normal skeletal muscle and lysosome-related gene deficiency induces accumulation of autophagosomes [9]. Autophagy regulates muscle homeostasis, removing protein aggregates and abnormal organelles that otherwise lead to muscle toxicity and dysfunction [10]. For instance, deficiency in the autophagy-related genes Atg5 or Atg7 is lethal in neonatal mice due to disruption of the supply of transplacental nutrients [10,11]. These findings suggest that autophagy deficiency plays a role in various forms of hereditary muscular dystrophy, including Bessler myopathy, Ullrich congenital muscular dystrophy, and Duchenne muscular dystrophy (DMD) [12]. Generally, muscle consists of multinucleated myofibers; myoblasts are mononuclear but fuse to form myotubes, and myoblasts can repair segmental loss of myofibers and fuse with existing fibers [13]. It is well known that the canonical Wnt signaling pathway is vital to regulate myoblast differentiation during skeletal muscle regeneration [14]. Dishevelled-2 (Dvl-2) is a key component of Wnt/β-catenin, which can be degraded by autophagy and further negatively regulates the canonical Wnt signal transduction [15].

In 2000, Davis et al. reported that myoferlin (MyoF) is upregulated in dystrophin-null muscle, thus implicating MyoF as a candidate gene in the pathogenesis of muscle dystrophy [16]. MyoF is a 230-kDa protein belonging to the ferlin family of proteins that also includes dysferlin and otoferlin. Ferlin family proteins share similar domain architecture, including a carboxy-terminal transmembrane domain and multiple amino terminal C2 domains [17]. Extensive studies have shown that MyoF is highly expressed in myoblasts and is essential in muscle cell functions such as plasma membrane integrity, myoblast fusion, and vesicle trafficking [18]. MyoF-deficient mice have been reported to display marked muscular dystrophy due to the unsuccessful fusion of myoblasts [19]. Studies in muscular dystrophy mice showed promoter-recapitulated normal MyoF expression was downregulated in healthy myofibers and was upregulated in response to myofiber damage, indicating that MyoF modulates muscle injury in both myoblasts and myofibers [20]. Based on the observation that the insulin-like growth factor 1 (IGF1) receptor accumulated in large vesicular structures in MyoF-null myoblasts, Demonbreun et al. concluded that MyoF is required for the IGF factor response and muscle growth [21]. However, other functions of the MyoF, such as its role in the regulation of muscle autophagy, remain to be clarified.

In this study, we investigated the function of MyoF in C2C12 myoblast differentiation and its role in muscular autophagy. We ascertained that MyoF interacts with Dvl-2 via the canonical Wnt signaling pathway. Taken together, our results provide a novel insight into the role of MyoF in autophagy.

## 2. Results 

### 2.1. MyoF is Highly Expressed in Differentiated Myogenic Cells

**Figure 1:**
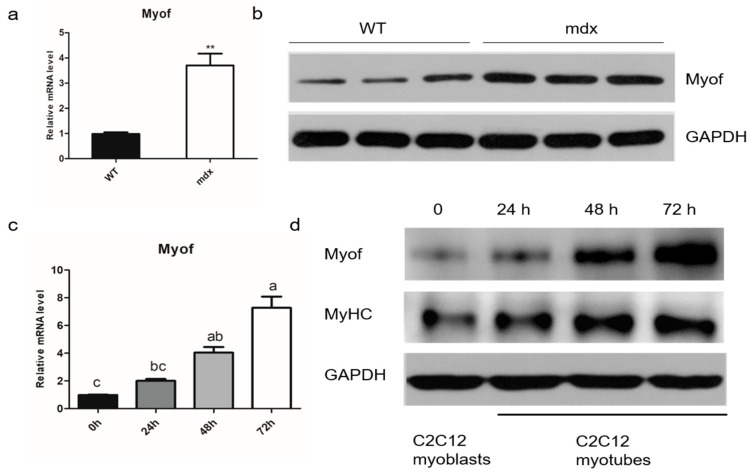
MyoF participates in adult skeletal muscle regeneration and myoblast differentiation. (**a**) Quantitative real-time PCR (qRT-PCR) analysis of MyoF expression in wild-type (WT) and mdx mice (aged 9 months); *n* = 3 per group. (**b**) Western blot analysis of MyoF protein levels in WT and mdx mice (aged 9 months); *n* = 3 per group. (**c**) qRT-PCR analysis of MyoF mRNA expression in C2C12 cells during differentiation. Bars not sharing the same letter labels are significantly different (*p* < 0.05; *n* = 3 independent cell cultures). (**d**) Western blot analysis of MyoF and MyHC protein levels during differentiation; GAPDH was used as loading control. Data represent means ± SEM (*n* = 3 independent cell cultures). * *p* < 0.05; ** *p* < 0.01.

Searches of the Gene Expression Omnibus (GEO) database showed that MyoF mRNA is highly expressed in mdx mice. We verified this information and found that MyoF was indeed upregulated in mdx mice, suggesting that MyoF is involved in skeletal muscle regeneration and repair (Figure 1a,b). To investigate the effects of MyoF on skeletal muscle development, we used the C2C12 myogenic cell line to mimic skeletal muscle differentiation in vitro. We found that MyoF expression increased gradually with myotubular differentiation of C2C12 cells. Increased MyoF expression was accompanied by increased myosin heavy chain (MyHC) expression during C2C12 myoblast differentiation (Figure 1c,d).

### 2.2. Role of MyoF in Skeletal Muscle Differentiation

**Figure 2:**
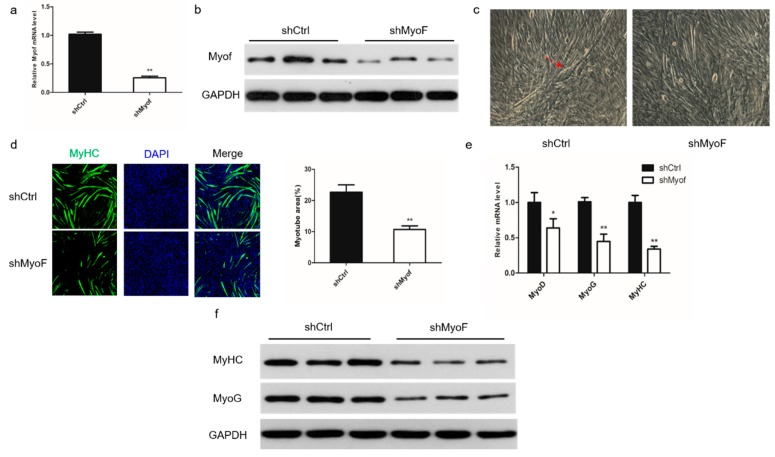
MyoF silencing reduces myoblast differentiation. C2C12 myoblasts were transfected with shMyoF or scramble plasmid (shCtrl) and expanded in growth medium or induced to differentiate into myotubes in differentiation medium. (**a**,**b**) qRT-PCR and Western blot analyses of MyoF mRNA and protein expression in MyoF-silenced and shCtrl cells, respectively; GAPDH was used as loading control. (**c**) Representative images of myotubes generated by cells transfected with shCtrl and shMyoF. (**d**) C2C12 cells transfected with MyoF-shRNA and shCtrl plasmid were cultured in differentiation medium for 72 h, then stained with MyHC antibody and DAPI (nuclei). The bar graph on the right shows the myotube area (%) after transfection with shRNA or shMyoF. (**e**,**f**) qRT-PCR and Western blot analyses of MyoD, MyoG, and MyHC mRNA and protein levels in MyoF-silenced and shCtrl cells, respectively; GAPDH was used as loading control. Data represent means ± SEM (*n* = 3 independent cell cultures). * *p* < 0.05; ** *p* < 0.01.

**Figure 3:**
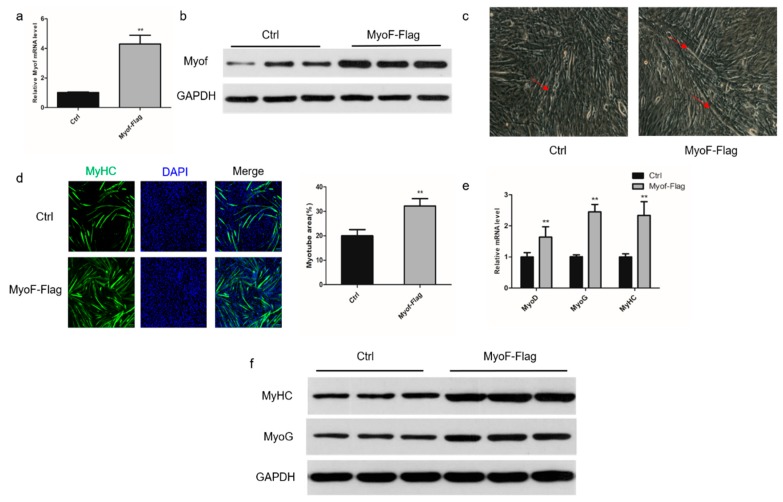
MyoF overexpression promotes myoblast differentiation. C2C12 myoblasts were transfected with the MyoF-Flag fusion protein plasmid or empty vector pcDNA3.1 (Ctrl), and were expanded in growth medium or induced to differentiate into myotubes in differentiation medium. (**a**,**b**) qRT-PCR and Western blot analyses of relative MyoF mRNA and protein expression levels at 72 h after transfection as described in the Materials and Methods, respectively; GAPDH was used as loading control. (**c**) Representative images of myotubes formed by Ctrl and MyoF-Flag cells. (**d**) Immunofluorescence staining of MyHC in C2C12 cells transfected with Ctrl or MyoF-Flag. The bar graph on the right shows the myotube area (%) after transfection with Ctrl or MyoF-Flag. (**e**) qRT-PCR analysis of MyoD, MyoG, and MyHC mRNA levels in Ctrl and MyoF-Flag cells. (**f**) Western blot analysis of MyHC and MyoG protein levels in Ctrl and MyoF-Flag cells; GAPDH was used as loading control. Data represent means ± SEM (*n* = 3 independent cell cultures). * *p* < 0.05; ** *p* < 0.01.

To investigate the role of MyoF during differentiation of C2C12 myoblasts, we silenced its expression by transfection with shRNA directed against MyoF (Figure 2a,b). Monitoring the morphological changes during differentiation showed a significant decrease in the total areas of myotubes, indicating that MyoF silencing impaired myoblast differentiation into myotubes (Figure 2c,d). In MyoF-silenced cells, expression of the myogenic regulatory factors, Myoblast determination protein 1 (MyoD), Myogenin (MyoG), and MyHC, was significantly reduced at the mRNA level (Figure 2e). Expression of MyoG and MyHC was also decreased at the protein level (Figure 2f). Additionally, MyoF overexpression promoted myotube formation and myogenic gene expression of the mRNA and protein levels (Figure 3a–f).

### 2.3. MyoF Rescues Skeletal Muscle Atrophy

**Figure 4:**
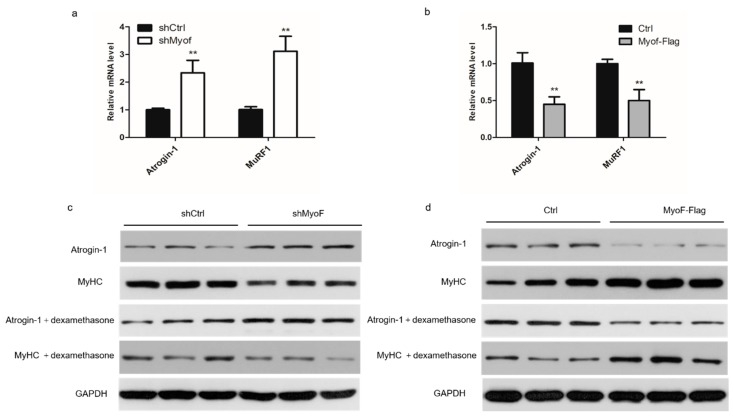
MyoF rescues skeletal muscle atrophy. Myotubes were treated with dexamethasone for 24 h to induce atrophy and then transfected with shRNA, shMyoF, or MyoF-Flag plasmid for 48 h. (**a**) qRT-PCR analysis of Atrogin-1 and MuRF1 mRNA levels in shCtrl and shMyoF cells. (**b**) qRT-PCR analysis of Atrogin-1 and MuRF1 mRNA levels in Ctrl and MyoF- Flag cells. (**c**) Western blot analysis of Atrogin-1 and MyHC protein expression in MyoF-silenced and shCtrl cells untreated or treated with dexamethasone. (**d**) Western blot analysis of MyHC and Atrogin-1 protein expression myotubes generated under treated or untreated dexamethasone induction of Ctrl and MyoF-Flag-transfected cells. GAPDH was used as loading control. Data represent means ± SEM (*n* = 3 independent cell cultures). * *p* < 0.05; ** *p* < 0.01.

We first studied the effect of MyoF on the expression of atrophy-related genes in myotubes. Myotubes transfected with shMyoF exhibited increased expression of two atrophy-related genes, Atrogin-1 and MuRF1 at the mRNA level, and Atrogin-1 at the protein level (Figure 4a,c). MyoF overexpression decreased expression of the two atrophy-related genes at the mRNA level and Atrogin-1 at the protein level (Figure 4b,d). We next investigated the ability of MyoF to rescue muscle atrophy by silencing or overexpressing MyoF during dexamethasone-induced myotube atrophy in vitro. In the presence of dexamethasone, MyoF silencing exacerbated the expression of atrophy-related genes in the myotubes at the protein levels (Figure 4c). Overexpression of MyoF attenuated the elevation of Atrogin-1 induced by dexamethasone (Figure 4d).

### 2.4. MyoF Functions by Dvl-2-Mediated Canonical Wnt Signaling

**Figure 5:**
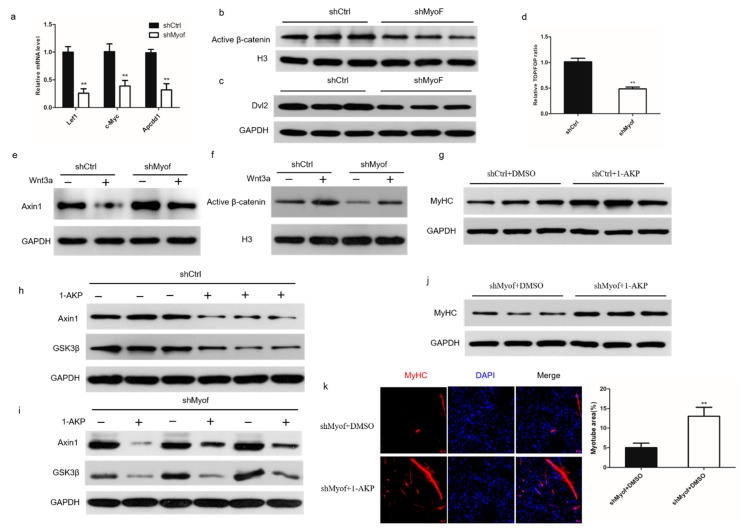
MyoF functions via Dvl-2-mediated canonical Wnt signaling. (**a**) qRT-PCR analysis of Wnt target gene (Lef1, c-Myc, and Apcdd1) expression in cells after transfection with shCtrl or shMyoF for 72 h. (**b**) Western blot analysis of β-catenin protein expression in nuclear lysates extracted from cells after transfection with shCtrl or shMyoF. (**c**) Western blot analysis of Dvl-2 protein expression. (**d**) TOP/FOP luciferase activity. (**e**) Western blot analysis of Axin1 protein levels after treatment with Wnt3a or saline for 24 h. (**f**) Western blot analysis of β-catenin protein levels in nuclear lysates extracted from cells after treatment with Wnt3a or saline for 24 h. (**g**) Western blot analysis of MyHC expression in shCtrl cells incubated with 1-AKP or DMSO for 72 h. (**h**) Western blot analysis of Axin1 and GSK3β protein levels in shCtrl-transfected cells after incubation with 1-AKP for 72 h. (**i**) Western blot analysis of Axin1 and GSK3β protein levels in shMyoF-transfected cells after incubation with 1-AKP for 72 h. (**j**) Western blot analysis of MyHC expression in shMyoF cells incubated with 1-AKP or DMSO for 72 h. (**k**) Immunofluorescence staining of MyHC in shMyoF cells incubated with 1-AKP or DMSO for 72 h, scale bars are 50 μm. The bar graph on the right shows the myotube area (%). Data represent means ± SEM (*n* = 3 independent cell cultures). * *p* < 0.05; ** *p* < 0.01.

Wnt/β-catenin signaling plays an important role in the control of myoblast differentiation. In our investigations of the role of MyoF in Wnt/β-catenin signaling, we found that expression of the Wnt target genes, lymphoid enhancer factor 1 (Lef1), MYC proto-oncogene (c-Myc), and APC downregulated 1 (Apcdd1), was significantly reduced in cells transfected with shMyoF (Figure 5a). Similarly, the level of active β-catenin in the nucleus was significantly reduced in MyoF-silenced cells compared to that in the control-transfected cells (Figure 5b). Western blot analysis also showed that Dvl-2 protein levels were significantly reduced in MyoF-silenced cells (Figure 5c). The TOP/FOP reporter assay also suggested that MyoF silencing significantly decreased Wnt signaling pathway activity (Figure 5d). Wnt family member 3a (Wnt3a), which is a classical ligand of the Wnt signaling pathway, activates the canonical Wnt signaling pathway [22]. We showed that Axin1 was degraded in control cells in the presence of Wnt3a. However, Axin1 still accumulated in MyoF-silenced C2C12 cells after Wnt3a treatment (Figure 5e). Furthermore, β-catenin translocation into the nucleus was significantly decreased in response to Wnt3a treatment in MyoF-silenced cells (Figure 5f). This indicated that MyoF is required for Wnt3a activation of the canonical l Wnt signaling pathway. To further investigate the mechanism by which MyoF activates the Wnt pathway, we used 1-azakenpaullone (1-AKP), which is known to activate the canonical Wnt signaling pathway independently of Dvl-2 [23]. We verified that 1-AKP can activate the Wnt signaling pathway and promote the expression of MyHC expression (Figure 5g,h). MyoF silencing reduced the levels of Axin1 and glycogen synthase kinase 3 beta (GSK3β) after the addition of 1-AKP (Figure 5i). Moreover, MyHC expression was increased following the addition of 1-AKP to MyoF-silenced cells (Figure 5j,k). These data showed that MyoF regulates myoblast differentiation via Dvl-2-mediated regulation of the canonical Wnt signaling pathway. 

### 2.5. MyoF Stabilizes Dvl-2 by Preventing Autophagy

**Figure 6:**
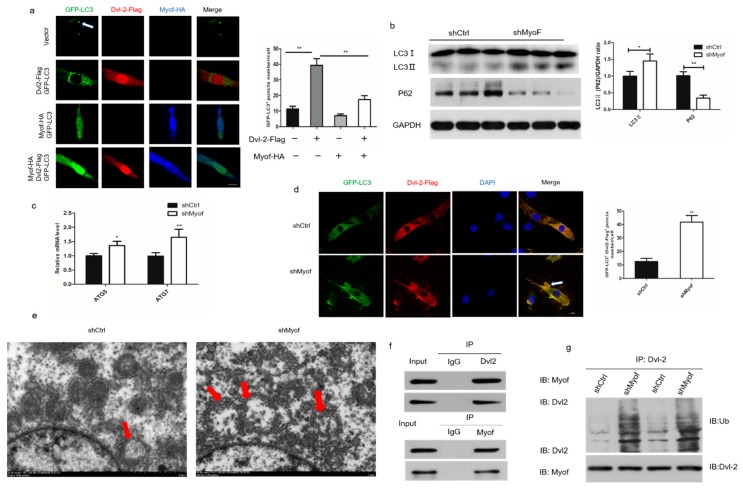
MyoF stabilizes Dvl-2 by antagonizing LC3-mediated autophagy. (**a**) Immunofluorescence analysis of GFP-LC3^+^ puncta in C2C12 cells after transfection with GFP-LC3 and Dvl-2-Flag plasmids, GFP-LC3 and MyoF-HA plasmids, or GFP-LC3, Dvl-2-Flag, and MyoF-HA plasmids. The bar graph on the right shows the number of GFP-LC3^+^ puncta in each cell. (**b**) Western blot analysis of p62 and LC3II protein levels in cells transfected with shCtrl or shMyoF. (**c**) qRT-PCR analysis of ATG5 and ATG7 mRNA levels in cells transfected with shCtrl and shMyoF. (**d**) Immunofluorescence analysis of GFP-LC3^+^/Dvl-2-Flag^+^ puncta in cells transfected with shCtrl or shMyoF. (**e**) The ultrastructure of cells transfected with shCtrl or MyoF observed by transmission electron microscopy (×5000). (**f**) Reciprocal coimmunoprecipitation analysis of MyoF and Dvl-2 in C2C12 cells. IB immunoblotting, IP immunoprecipitation. (**g**) Western blot analysis of the Dvl-2 ubiquitination levels in cells transfected with shCtrl or shMyoF. Data represent means ± SEM (*n* = 3 independent cell cultures). * *p* < 0.05; ** *p* < 0.01.

Autophagy attenuates Wnt signaling by inducing Dvl-2 degradation. Thus, we investigated the influence of MyoF expression in skeletal muscle on autophagy induction. Expression of Dvl-2-Flag alone resulted in promotion of GFP-LC3 puncta formation in C2C12 cells. However, cotransfection with MyoF-HA and Dvl-2-Flag significantly reduced LC3 puncta formation in C2C12 cells compared to that observed following transfection with Dvl-2-Flag alone (Figure 6a). In addition, Western blot showed that MyoF silencing increased LC3II expression and decreased p62 expression at the protein level (Figure 6b). We observed a significant increase in mRNA expression of ATG5 and ATG7 in MyoF-silenced cells compared to that in control-transfected cells (Figure 6c). Immunofluorescence analysis also revealed a significantly increased number of LC3 puncta in MyoF-silenced cells (Figure 6d). Electron microscopy showed a significant increase in the number of autophagosomes in MyoF-silenced cells compared to that in control-transfected cells (Figure 6e). Immunofluorescence analysis showed that MyoF and Dvl-2 were uniformly distributed in C2C12 cells. In further investigations of the interaction between MyoF and Dvl-2 in the antagonistic autophagy system, immunoprecipitation analysis showed that MyoF interacts with Dvl-2 (Figure 6f). Moreover, the levels of Dvl-2 ubiquitination were significantly increased in MyoF-silenced cells compared to those in control-transfected cells (Figure 6g). Collectively, these results indicate that MyoF interacts with Dvl-2 to facilitate its resistance to ubiquitination, and thus prevent the autophagy process.

## 3. Discussion

Although MyoF has been proven to be the pathogenic gene of muscular dystrophy, its antagonism against autophagy by stabilizing Dvl-2 has not yet been determined. In this study, we found that MyoF is highly expressed in mdx mice and participates in the growth of C2C12 cells, which is in accordance with the report of the unique function of MyoF in muscle regeneration and degeneration in muscular dystrophies [24]. MyoF is widely regarded as a muscle-specific protein. The growth of skeletal muscle is a multistep process accompanied by myoblast fusion to form myotubes, a process in which MyoF functions in the maturation of myotubes [25]. Our results showed that the normal C2C12 cells’ development was retarded when there was interference with shMyoF, while conversely, overexpression of MyoF facilitated the growth of C2C12, which indicated that MyoF plays a role in promoting C2C12 differentiation. MyoF expression increases markedly when myoblasts undergo fusion. However, the function of MyoF may depend on cooperation with other molecules. Doherty et al. showed that the second C2 domain of MyoF interacts with EHD2 and the combination regulates normal myoblasts membrane fusion [26]. Furthermore, MyoF not only regulates myoblast fusion, but also the formation of transverse tubules and responses to muscle injury in both myoblasts and mature myofibers [27]. Taken together, these reports demonstrate that MyoF plays a vital role in the growth and development of skeletal muscle, although its specific molecular mechanism remains to be fully elucidated.

Muscle atrophy occurs under conditions such as injury, denervation, glucocorticoid treatment, sepsis, and aging [28]. The physiology of muscle atrophy appears as a loss of tension, and there is substantial evidence that a reduction in protein synthesis and enhanced protein degradation contribute to muscular atrophy [29]. The complex molecular signaling events underlying atrophy are not fully understood. In our study, we performed MyoF knockdown and overexpression in C2C12 cells to explore the role of MyoF in muscle atrophy. Our results showed that the protein abundance of Atrogin-1 and MyHC was affected by MyoF knockdown, overexpression, and dexamethasone treatment. Since Atrogin-1 is a marker gene of muscle atrophy, these results indicate that MyoF has the potential to rescue muscle atrophy. It has been reported that MyoF is upregulated in muscle undergoing repeated degeneration and regeneration and MyoF is regarded as a candidate gene involved in the pathogenesis of muscle dystrophy [16]. In spite of extensive research on muscle disease, little is known about the role of MyoF in muscular atrophy, although MyoF may be a modifier.

The canonical Wnt signaling pathway is widely reported to impact all aspects of skeletal muscle, including myogenetic lineage and proliferation of cells [30]. Key proteins in the canonical Wnt signaling pathway, such as Wnt1, Wnt3a, and Wnt5a, regulate proliferation of skeletal muscle satellite cells during injury healing [31]. A wealth of recent data show that the canonical Wnt is targeted and activated to regulate myoblast proliferation. R-spondin1 has been shown to mediate reciprocal control of the canonical Wnt signaling pathway in muscle stem cell progeny to ensure muscular tissue repair after wounding [32]. Furthermore, the canonical Wnt has been shown to promote differentiation in skeletal muscle positively regulated by HDAC8 [33]. In our study, MyoF silencing had marked effects on the expression of Lef1, c-Myc, Apcdd1 and active β-catenin, and Dvl-2, the Wnt target genes and node proteins. Further investigations showed that MyoF silencing disturbed the canonical Wnt pathway in C2C12 by upregulating Axin 1. Previous studies confirmed that Axin 1 mediates the disassembly of β-catenin structure by promoting its phosphorylation catalyzed by GSK-3β [34]. Therefore, we hypothesized that MyoF plays a vital but indirect role in controlling skeletal muscle development via the canonical Wnt signaling pathway. Dvl contains three highly conserved domains, termed Dvl-1, Dvl-2, and Dvl-3, and is expressed ubiquitously throughout development [35]. In the canonical Wnt pathway, Dvl-2 mediates the integration of the receptors and a destruction complex to induced β-catenin degradation, after which β-catenin is translocated into and accumulates in the nucleus, where it interacts with T cell-specific factor/LEF to initiate Wnt target gene transcription [36]. Dvl-2 plays a role in the upstream of the Wnt signal transduction pathway of β-catenin and GSK-3β, and can positively regulate the Wnt signal pathway [34]. 1-AKP is a selective inhibitor of GSK-3β, which results in GSK-3β not being able to form complex with beta-catenin in collaboration with APC and Axin, leading to the accumulation of β-catenin in the cytoplasm and the introduction of large amounts of β-catenin into the nucleus, thus requiring no direct activation of the Wnt signaling pathway by Dvl-2 [21]. Autophagy is a lysosome-dependent degradation pathway that exists widely in eukaryotic cells and is regulated by cell signaling pathways, such as the canonical Wnt pathway. In 2010, Gao et al. demonstrated that ubiquitinated Dvl-2 is recognized by p62, resulting in the formation of a large aggregate composed of p62 and LC3, which then selectively induces autophagy and degradation via the lysosomal pathway [37]. These findings illustrated that autophagy cooperates negatively with the canonical Wnt pathway by inverse regulation of Dvl-2. Indeed, a rich set of data have confirmed that Dvl-2 degradation is negatively regulated by the autophagy signaling pathway. Recent studies have shown that the Wnt pathway is inhibited and Dvl-2 degradation enhanced by GABARAPL1 via autophagy signaling [38,39]. It has also been reported that autophagic degradation of Dvl-2 is restrained by IRS1/2, which interacts and forms a complex with Dvl-2. Thus, IRS1/2 positively controls Wnt/β-catenin signaling via Dvl-2 [36]. In our study, we observed that cotransfection of C2C12 cells with MyoF and Dvl-2 attenuated autophagic degradation and thus, we speculated that MyoF is involved in the regulation of Dvl-2. Indeed, our data suggest that MyoF interacts with Dvl-2 protein to regulate muscle development through regulation of autophagy via the canonical Wnt signaling pathway.

**Figure 7:**
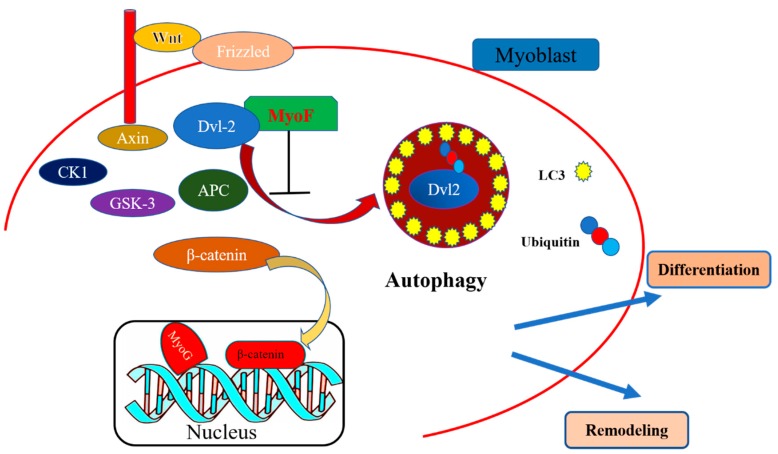
Model of the role of MyoF during skeletal muscle development. MyoF interacts with Dvl-2 to antagonize autophagy-mediated degradation, thereby activating the Wnt/β-catenin signaling pathway to regulate skeletal muscle development.

In conclusion, our study shows that MyoF regulates the canonical Wnt signaling pathway by stabilizing Dvl-2 to downregulate its autophagic degradation (Figure 7). These findings extend our understanding of the molecular mechanism by which MyoF is involved in skeletal muscle development and shed light on the role of MyoF in the alleviation of muscular autophagy mediated by the canonical Wnt signaling pathway.

## 4. Materials and Methods 

### 4.1. Cell Cultures

The C2C12 mouse myoblast cell line was purchased from FuHeng Cell Center (FuHeng, Shanghai, China) and expanded in growth medium (Dulbecco’s modified Eagle’s medium, DMEM; Sigma, MO, USA), supplemented with 10% fetal bovine serum (Gibco, Grand Island, NY, USA), and 1% antibiotic-antimycotic (ABAM) (Solarbio, Beijing, China)] at 37 °C under 5% CO_2_ in air. When cells reached 90% confluence, myotube differentiation was induced by cultivation in differentiation medium containing 2% horse serum (Hyclone, Logan, UT, USA), and 1% ABAM.

### 4.2. MyoF Knockdown and Overexpression

For overexpression or silencing of MyoF in C2C12 myoblasts, cells were seeded into 6-well plates and transfected with a pcDNA3.1 expression vector encoding MyoF-Flag or mouse MyoF-shRNA 2 μg, respectively, using Lipofectamine 3000 (Invitrogen, Carlsbad, CA, USA) according to the manufacturer’s instructions. MyoF was detected by Western blot and quantitative qRT-PCR analyses.

### 4.3. RNA Extraction and Real-Time PCR

Cells were washed twice with phosphate buffer saline (PBS), and total RNA was extracted from with TRIzol reagent (Takara, Tokyo, Japan) according to the manufacturer’s instructions. Quantitative RT-PCR was performed according to a previously described method [40]. All amplicon primer sets were designed using the Sangon Biotech Primer Design Center (Shanghai, China); details of the primers used are shown in Table 1. Gene expression was determined using average cycle thresholds normalized to GAPDH according to the 2^−ΔΔCT^ method [41].

### 4.4. Cell Treatment Protocols and Antibodies

The following primary antibodies were used in this study: mouse anti-MyHC (Santa Cruz, Heidelberg, Germany), mouse anti-MyoG (Sigma), rabbit anti-Atrogin-1 (Novus Biological, Abingdon, UK), rabbit anti-Ubiquitin (Sigma), rabbit anti-LC3B (Sigma), rabbit anti-MyoF ((Novus Biological), rabbit anti-Axin-1 (Sigma), rabbit anti-GSK3β (Abcam, Cambridge, UK), rabbit anti-β-catenin (Sigma), rabbit anti-Dvl-2 (Abcam), rabbit anti-p62 (Sigma), rabbit anti-LC3B (Sigma), rabbit anti-GAPDH (Santa), and rabbit anti-Histone H3 (Abcam). The following secondary antibodies were used: mouse anti-rabbit (Santa Cruz), goat anti-rabbit (Santa Cruz), mouse anti-rabbit horseradish peroxidase, goat anti-mouse HRP, and donkey anti-goat HRP (Sigma). Myotube atrophy was induced by treatment with 10 μM dexamethasone (Sigma) for 24 h.

### 4.5. Western Blot and Immunoprecipitation (IP) Analysis

For Western blot analysis, cells were washed with PBS and lysed in RIPA lysis buffer (Bioss, Beijing, China). Next, total protein (200 μg) was separated by 12% SDS-polyacrylamide gel electrophoresis (SDS-PAGE), and transferred to a polyvinylidene fluoride (PVDF) membrane (Millipore Corporation, Billerica, MA, USA). The PVDF membrane was blocked with 5% nonfat milk at room temperature for 1 h, followed by incubation with the appropriate specific primary antibodies overnight at 4 °C. The PVDF membrane was then rinsed with Tris-Buffered Saline Tween-20 (TBST) and stained with the appropriate horseradish peroxidase (HRP)-labeled secondary antibody for 1 h at room temperature. After washing with TBST, proteins were visualized with Electrochemiluminescence (ECL) reagent (Amersham Pharmacia Biotech, Piscataway, NJ, USA).

For immunoprecipitation analysis, the cells were lysed with IP lysis buffer, and the total protein (5 μg) was immunoprecipitated with anti-MyoF and anti-Dvl-2 antibodies. Immunocomplexes were washed three times with IP lysis buffer and analyzed by Western blotting as described. Quantification of protein blots was performed with the Quantity One 1-D software (version 4.4.0) (Bio-Rad, Hercules, CA, USA) using images acquired from an EU-88 image scanner (GE Healthcare, King of Prussia, PA, USA).

### 4.6. Immunofluorescence and Confocal Microscopy

Cells grown on coverslips were rinsed in PBS and fixed with 4% paraformaldehyde (Solarbio) for 10 min. After fixation, cells were washed twice with PBS and permeabilized with 0.2% Triton X-100 for 10 min, washed with PBS, and incubated with the relevant antibodies diluted in PBS/10% FSC for 1 h. The cells were then rinsed three times with PBS for 5 min each time. After incubation with the relevant primary antibody, cells were washed and incubated with the fluorescence-labeled secondary antibody for 1 h at room temperature in the dark. Subsequently, the cells were washed three times with TBST and fluorescence intensity was observed with an Olympus FluoView FV1000 confocal microscope (Olympus, Melville, NY, USA).

### 4.7. Transmission Electron Microscopy

Cells were scraped gently from culture plates and washed twice with PBS. The cells were then fixed in 2.5% glutaraldehyde PBS for 15 min, and postfixed in 1% osmium tetroxide for 2 h at room temperature. After washing three times in distilled water, the cells were exposed to 1% uranylacetate for 15 min. The samples were dehydrated in a graded ethanol series and embedded in Spurr’s low-viscosity media. Ultrathin sections (80 nm) were prepared stained with uranyl acetate and lead citrate, and observed using JEM-1400 TEM (JEOL, Tokyo, Japan). Images were captured using a CCD camera AMT (Sony, Tokyo, Japan).

### 4.8. Statistical Analysis

All statistical analyses were performed using SPSS 17.0 (SPSS Inc., Chicago, IL, USA). Data are presented as least squares means ± standard error of the mean (SEM), and values were considered statistically different at *p* < 0.05.

## Figures and Tables

**Table 1:** Primers used for quantitative real-time PCR analysis.

Genes	Forward Primer (5′-3′)	Reverse Primer (5′-3′)
MyoF	TGCCACTAACATCCCTAA	ATCACCACCGATTCTACTC
MyoD	AGCACTACAGTGGCGACTCA	GGCCGCTGTAATCCATCA
MyoG	TACAGCGACCAACAGTACGC	TCTGCATTGTTTCCATCCTG
MyHC	CGGCTGCCTAAAGTGGAGAT	AGGCCTGTAGGCGCTCAA
Atrogin-1	GCAAACACTGCCACATTCTCTC	CTTGAGGGGAAAGTGAGACG
MuRF1	ACCTGCTGGTGGAAAACATC	CTTCGTGTTCCTTGCACATC
Apcdd1	CTGAAGCATCTCCACAACGG	GGACCCGACCTTACTTCACA
c-Myc	TAGTGCTGCATGAGGAGACA	CTCCACAGACACCACATCAA
Lef1	GACAGATCACCCCACCCATT	ATAGCTGGATGAGGGATGCC
ATG5	AGCAGCTCTGGATGGGACTGC	GCCGCTCCGTCGTGGTCTGA
ATG7	GCTCCTCATCACTTTTTGCCAACA	GGAGCCACCACATCATTGC
GAPDH	GTGCCGCCTGGAGAAACCT	AAGTCGCAGGAGACAACC
